# Outlook on zero/ultrashort echo time techniques in functional MRI

**DOI:** 10.1002/mrm.70065

**Published:** 2025-09-06

**Authors:** Silvia Mangia, Shalom Michaeli, Olli Gröhn

**Affiliations:** 1 Center for Magnetic Resonance Research, Department of Radiology, University of Minnesota, Minneapolis, Minnesota, USA; 2 A.I. Virtanen Institute, University of Eastern Finland, Kuopio, Finland

**Keywords:** awake animals, body motion, BOLD, fMRI, human brain, susceptibility artifacts, UTE, zero-TE

## Abstract

Since its introduction more than 30 years ago, the blood oxygenation level dependent (BOLD) contrast remains the most widely used method for functional MRI (fMRI) in humans and animal models. The BOLD contrast is typically acquired with echo planar imaging (EPI) to obtain sensitization of the signal during the echo time (TE) to dynamic changes in deoxyhemoglobin content, while achieving high spatiotemporal resolution and full brain coverage. However, EPI-based fMRI also faces multiple shortcomings, including sensitivity to body motion, susceptibility-related signal dropouts, interference with multimodal sensors, and loud acoustic noise. Here we provide a succinct overview and outlook of alternative strategies for fMRI relying on free induction decay–based techniques, which, by using zero/ultrashort TE, inherently solve most of these challenges. Such approaches are receiving increasing attention in the field of fMRI, motivated by initial findings in humans and animal models in which robust functional contrast was obtained despite the absence of an echo, primarily via sensitization to inflowing blood. We therefore discuss the benefits and current shortcomings of zero/ultrashort TE fMRI versus conventional EPI-based fMRI, the opportunities for enabling fMRI designs that are challenging with EPI-based approaches, and the state of progress toward use in clinical settings. Overall, zero/ultrashort TE fMRI is predicted to become a powerful new tool for basic, clinical, and preclinical research, especially for applications at ultrahigh magnetic fields, studies in awake animals, multimodal imaging, investigations requiring minimization of scanning noise, and fMRI beyond the brain.

## 1 | INTRODUCTION

The impact that the blood oxygenation level dependent (BOLD) contrast for functional MRI (fMRI) has had on neuroscience cannot be overstated. Since Ogawa’s 1990 discovery of T_2_* and T_2_ signal contrasts from deoxygenated blood in rat brains and phantoms,^1,2^ and subsequent human applications,^3–5^ BOLD-fMRI has become a revolutionary tool to map brain function and connectivity noninvasively,^6,7^ with sensitivity and specificity benefits offered by ultrahigh magnetic fields.^8^ Although gradient-echo or spin-echo echo planar imaging (EPI) detect the alterations in deoxyhemoglobin content resulting from neurometabolic and neurovascular coupling events, other T_1_-mediated EPI-based schemes can be used for sensitizing the contrast selectively to cerebral blood flow (CBF)^9–11^ or volume (CBV).^12,13^

Despite the wide application of EPI-based fMRI, there have been continuing efforts to develop alternative methods. Most popular, albeit at times controversial, are methods for detecting neuronal activity more directly than with metabolic/hemodynamic outcomes.^14–20^ Here, we focus on the lesser-known quest for alternative methods capable of mitigating the technical obstacles of EPI-based fMRI. In fact, although EPI time series can be acquired at very high spatial and temporal resolutions,^21–26^ they can be affected by signal dropouts and image distortions due to susceptibility artifacts induced by air–tissue interfaces and by metallic implants, and they are highly sensitive to body motion. Furthermore, the fast gradient switching in EPI interferes with electrophysiological recordings, resulting in large artifacts that require special postprocessing. The fast gradient switching also produces scanner vibrations and acoustic noises often well above >100 dB, which is problematic for subject comfort and whenever a quiet environment is compulsory.

To establish methods that solve the aforementioned challenges of EPI-based fMRI, free induction decay (FID)–based approaches have been proposed for mapping brain activity in absence of deoxyhemoglobin-related T_2_ or T_2_* effects.^27,28^ FID-based techniques include several zero-TE variants, such as zero echo time (ZTE) imaging,^29–31^ PETRA (pointwise encoding time reduction with radial acquisition),^32^ sweep imaging with Fourier transformation (SWIFT),^33^ Multi-Band SWIFT (MB-SWIFT),^34^ and SORDINO (steady-state on-the-ramp detection of induction decay with oversampling),^35^ along with ultrashort echo time (UTE) imaging.^36^ Importantly, the main difference among zero-TE and UTE sequences is in how gradients are used, leading to incremental gradient switching in zero-TE versus large gradient steps in UTE. As discussed below and summarized in Table 1, FID-based approaches are receiving increasing attention in fMRI owing to initial promising results, even though their translation to clinical systems is currently a partially unfilled goal.

## 2 | DEMONSTRATION AND INTERPRETATION OF FUNCTIONAL SIGNAL CONTRAST

**FIGURE 1:**
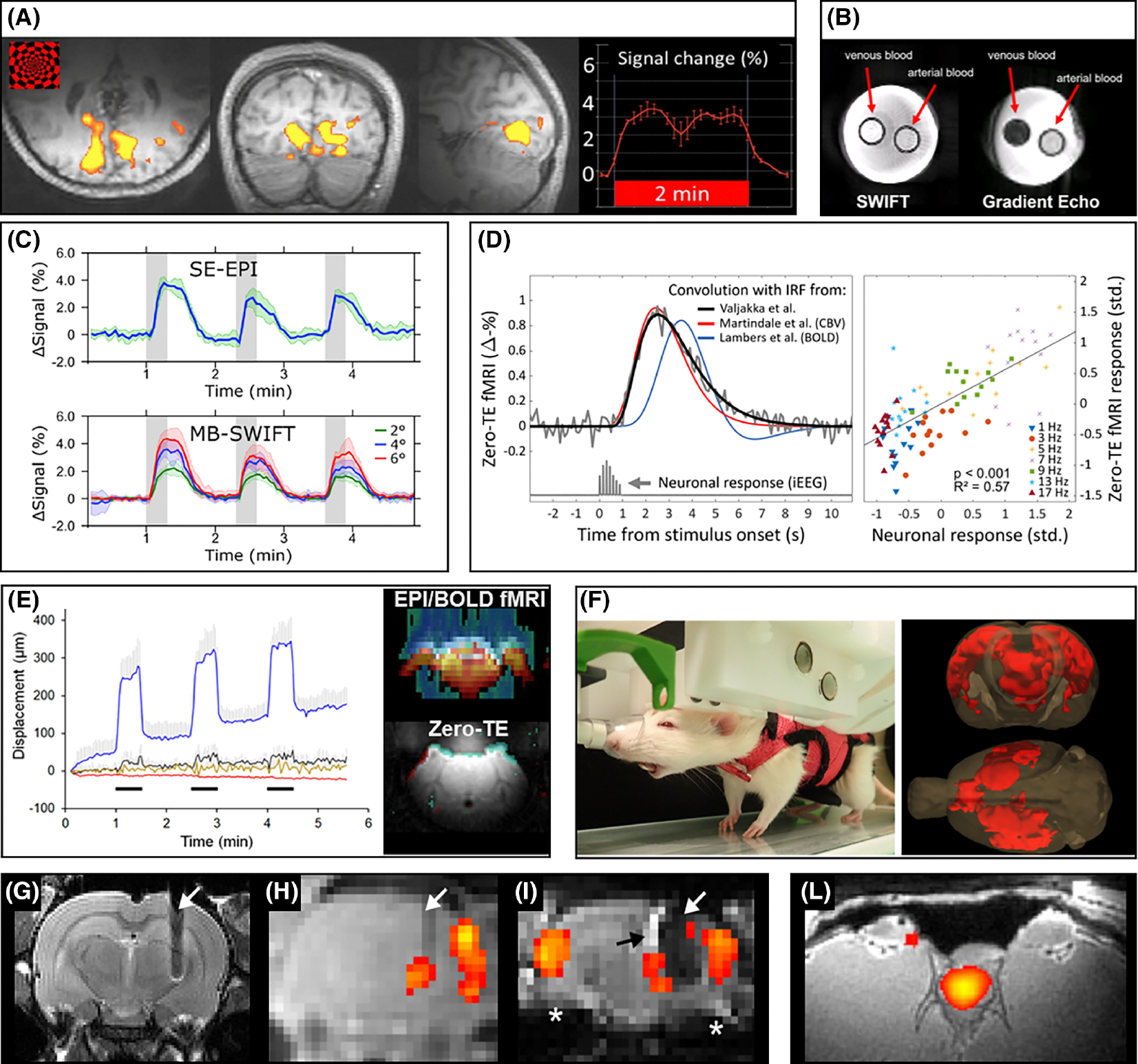
Initial demonstration, features, and setups of free induction decay (FID)–based functional MRI (fMRI). (A) Sweep imaging with Fourier transformation (SWIFT) fMRI in humans. Activation maps obtained with SWIFT (repetition time=5.2 s) during a visual stimulation paradigm from one representative subject; threshold *p* < 3.3e−10. (B) SWIFT contrast in phantoms. 5-mm tube samples containing venous/arterial blood, imaged with SWIFT or gradient recalled echo at 9.4 T. Panels (A) and (B) and relative captions are from ISMRM Proceedings^27^ under CC-BY license. (C) Dependence of Multi-Band SWIFT (MB-SWIFT) functional contrast on flip angle. Mean time series measured during deep brain stimulation in the rat ipsilateral somatosensory cortex (*n* = 6), as detected by spin-echo echo planar imaging (SE-EPI; *top*) and MB-SWIFT (*bottom*) with different flip angles.^28^ (D) Temporal features of zero-TE functional contrast and correlation with electrical activity measured with intracranial EEG (iEEG) during electrical whisker pad stimulation.^41^ On the left, zero-TE response (*gray*) is shown along with the iEEG response convolved with the impulse response function (IRF) determined for zero-TE fMRI (*black*), for cerebral blood volume fMRI (CBV; *red*)^50^, and for blood oxygen level dependent fMRI (BOLD; blue)^51^; on the right, correlation of neuronal and fMRI responses are shown for different frequencies of whisker stimulation (each data point represents the mean response in one subject at one stimulation frequency). (E) Resilience of zero-TE fMRI to body motion. On the left, signal changes caused by controlled body movement of head fixed rats (*n* = 6) determined as displacement: uncorrected EPI (*blue*), EPI with movement correction (*black*), EPI with movement correction and regression (*brown*), and zero-TE fMRI without any movement correction (*red*). On the right, corresponding general linear model analysis in one presentative rat using periods of body movements as “activation periods” demonstrates superior tolerance of zero-TE approach (MB-SWIFT in this case) to body movement.^37^ (F) MB-SWIFT fMRI in awake, behaving rats. Setup of minimally restrained rats expressing behavior, and activation of cortico-thalamic somatomotor areas and the medial frontal cortex of one presentative rat during retreat attempt (*n* = 19 attempts).^52^ (G–I) MB-SWIFT fMRI around implanted leads. A tungsten deep brain stimulation electrode visualized by fast spin-echo (G) has minimal artifacts in MB-SWIFT fMRI (H), whereas in spin-echo EPI fMRI (I) it has a large signal void area (*white arrow*) and signal pile-up (*black arrow*) caused by electrode and significant artifacts from tissue air interface (*asterisk*). Results demonstrate very high tolerance of MB-SWIFT to susceptibility induced B_0_ inhomogeneity when compared with EPI.^28^ (L) MB-SWIFT fMRI in spinal cord. Activation map measured in the spinal cord of one representative rat during epidural electrical stimulation demonstrates high sensitivity of fMRI detection and distortionless images, which would not be possible with EPI-based fMRI.^40^ Panels (C)–(L) and captions are reproduced or modified from open access publications^28,37,40,41,52^ under CC-BY or CC-BY-NC-ND license.

The first proof-of-principle demonstration of an FID-based approach (SWIFT) for fMRI was obtained in the human visual cortex with a local transmit/receive (T/R) radiofrequency (RF) coil on a research 4T scanner.^27^ SWIFT was shown to produce robust task-evoked signals (Figure 1A) of comparable amplitude to those observed with gradient-echo EPI, leading to similar functional signal-to-noise ratio (SNR). Phantom studies provided evidence that the functional contrast does not originate from blood oxygenation (Figure 1B),^27^ whereas later studies in rodents highlighted that the functional contrast depends on the flip angle (Figure 1C) and is thus T_1_-mediated.^28^ Moreover, the temporal features of the functional response are consistent with a hemodynamic origin of the contrast, and the fMRI signals are well predicted by electrophysiological recordings (Figure 1D).^41^ Our current understanding is that most of the FID-based functional contrast originates from inflow of unsaturated blood into the brain during local RF transmission, leading to higher magnetization of blood than that of parenchyma and, ultimately, to positive signal changes when CBF and CBV increase. Additional T_1_ contributions conceivably originate from changes in molecular oxygen,^35^ although their relative contributions remain to be firmly established. Other mechanisms as neuronal and/or glial swelling,^53^ changes in arterial pressure,^54^ and mobility changes of water molecules at the interface of excitable cell membranes, are possible but likely small.

## 3 | BENEFITS AND CURRENT SHORTCOMINGS

FID-based sequences are typically used for anatomical imaging of short-T_2_ targets; however, they also enable full brain coverage with spatial and temporal resolutions suitable for fMRI. The zero/ultrashort TE used in FID-based sequences intrinsically minimizes spin dephasing originating from B_0_ variations. FID-based sequences further tolerate B_0_ variations by achieving high excitation bandwidth via the short excitation pulses, and fast encoding with high readout bandwidth in all spatial directions via the radial readout, in which case ultrafast T/R switches are also recommended to minimize loss of initial FID-points that lead to truncation artifacts. Therefore, FID-based techniques effectively reduce sensitivity to B_0_ variations such as those induced by body motion (Figure 1E)^35,37^ and susceptibility gradients.^28,35,55^ In addition, zero-TE sequences use incremental gradient switching, which largely resolves gradient-induced artifacts in electrical recordings^37^ and produces a nearly silent, continuous acoustic noise that increases the comfort of the subject.^35,37^ Perhaps the most compelling application so far has been the detection of whole-brain activity in head-fixed awake rats^37,48^ and mice,^56^ allowing preclinical fMRI studies in minimally restrained animals that express behavior (Figure 1F)^35,52^ and potentially during natural sleep. In stark contrast to EPI-based fMRI, the resilience of FID-based fMRI to B_0_ inhomogeneities not only removes the need of dedicated B_0_ shimming, but it also benefits the monitoring of brain activity around implanted leads (Figure 1G–I)^28,38,39^ and of spinal cord activity during epidural electrical stimulation (Figure 1L).^40^ A novel dual-FOV MB-SWIFT configuration even enables simultaneous fMRI of rat brain and spinal cord,^57^ or potentially two brains for social MRI. With proper spoke resampling,^41,43^ FID-based fMRI can reach ultrahigh temporal resolution of 100 ms and below for recurring events—a feature that may be exploited for pulsatility imaging^44^ or for putative direct imaging of neuronal activity^20^ in the whole brain. Zero-TE readouts can be also combined with T_2_ or T_2_* preparations to achieve quiet BOLD fMRI,^58,59^ although in this case they do not fully mitigate susceptibility and body motion artifacts due to the echo formation.

Because the inflow-enhanced CBF/CBV contrast in FID-based fMRI depends on coil geometry, flip angle, vascular anatomy and blood velocity, optimizations are needed for maximizing functional contrast. This task can be easily achieved when benefiting from the flexibility of hardware solutions and software programming available on research-oriented scanners, as proven by the increasingly mature FID-based fMRI applications in animal studies.^28,35,37–39,41,43,52,55^ Yet, clinical translation of FID-based fMRI has been challenging, with only preliminary human applications being published so far.^27,49^ The main obstacle has been limited access to local T/R coils and to proper hardware components that enable ultrafast T/R switching, and lack of off-the-shelf sequences that are readily programmable to become fMRI-suitable. However, development of hardware solutions on human systems is a focus of current efforts.^60,61^ Software solutions are also possible, although tradeoffs are unavoidable. For instance, although not acoustically quiet, UTE can be sensitized to inflow with minor sequence modifications that lead to similar functional SNR to that of BOLD-fMRI,^46^ enabling investigations even in body regions previously precluded to functional neuroimaging such as the nasal cavities.^47^

## 4 | CONCLUDING REMARKS

As FID-based fMRI progresses, there will be numerous opportunities for advancing acquisition/processing solutions to improve image quality, sensitivity, and spatiotemporal resolution. In particular, we expect FID-based fMRI to address research questions in settings that are challenging with EPI, especially at ultrahigh magnetic fields.^42^

## Figures and Tables

**TABLE 1:** State of the art of zero/ultrashort TE fMRI versus EPI-based fMRI.

Feature	EPI-based strategies	Zero/ultrashort TE strategies
**A. General characteristics of acquisition schemes**		
Readout type	• 2D or 3D • Typically Cartesian • TE in the order of tens of ms	• Zero-TE approaches are 3D; UTE can be 2D or 3D • Radial or variants of center-out readouts • Zero-TE approaches have no encoding delays because excitation happens in the presence of gradients; acquisition starts at the end of the excitation pulse, after an intrinsic T/R switch delay; the missing FID points lead to truncation artifacts, which are more pronounced if high readout BWs are desired, requiring special postprocessing or separate acquisition (PETRA^32^). On research scanners, T/R switches are typically ultrafast, with T/R switch delays being a few gs; T/R switch delays are tens of gs on clinical scanners • In UTE, excitation happens in the absence of gradients, therefore no encoding occurs during the pulse; acquisition starts after the excitation pulse (after a T/R switch delay) together with the ramping of the encoding gradient, therefore the “echo time” in UTE cannot be zero, and it effectively depends on the ramp-up time, typically in the order of 100 gs on clinical scanners • Zero-TE approaches lack slice/slab selection, which can restrict the size of the FOV and the spatial resolution
Scheme variants	• GRE-EPI and SE-EPI, to obtain BOLD contrast (for review, see Ref. 26) • Arterial spin labeling, to obtain CBF contrast (for reviews, see Refs. 10,11) • Vascular space occupancy (VASO), to obtain CBV contrast^12^ • Single-shot, multishot EPI; multiband EPI, etc., (for reviews, see Refs. 7,23)	• FID-based approaches include: ZTE: hard-pulse excitation followed by no-acquisitiondelay readout during constant gradients with small stepwise increments for sampling different spokes; gradient spoiling is optional after each FID acquisition^31^ PETRA: similar to ZTE, but it adds a separate scan to acquire missing FID points with pointwise encoding^32^ SWIFT: frequency- and amplitude-modulated excitation gapped with no-acquisition-delay readouts during constant gradients with small stepwise increments for sampling different spokes; gradient spoiling is optional after each FID acquisition^33^ MB-SWIFT: multiple-frequency hard-pulse excitations followed by no-acquisition-delay readouts during each constant gradient [spokes] to achieve high excitation and readout bandwidth; gradient spoiling is optional after each FID acquisition^34^ SORDINO: hard-pulse excitation followed by no-acquisition-delay readout during constantly changing gradients [curved spokes]; only RF, no gradient spoiling^35^ UTE: excitation pulse applied without presence of a readout gradient, followed by ramping encoding gradient (ramp-up time typically 100 gs) while starting acquisition; allows slab selection unlike zero-TE approaches^36^
Sensitivity to B_0_ inhomogeneities	• High sensitivity to B_0_ inhomogeneities, induced by, e.g., susceptibility gradients, metallic implants, body motion, respiration • Frequency shifts (e.g., due to fat) appear as shifts in the image space; the effect can be minimized with multishot acquisitions • Motion affects the entire k-space trajectory of the slice acquisition during which the motion event happened; the effect appears as image distortions or ghosting	• As compared with EPI, negligible sensitivity to B_0_ inhomogeneities, induced by, e.g., susceptibility gradients, metallic implants, body motion, respirations^37^ • Frequency shifts (e.g., due to fat) appear as blurring in the image space; the effect can be minimized by increasing the FOV bandwidth • Motion affects only those spokes in k-space during which the motion event happened, with less impact on image quality as compared with EPI; the effect may appear as under-sampling strikes and/or blurring
Sensitivity to non-tissue signal sources	• None	• Short-T_2_ signals from non-tissue sources (e.g., plastic of coil holders, tubing) can be a confounding factor, especially if originating from outside the FOV
Impact of gradient switching and duty cycle	• Fast gradient switching can induce eddy currents, potentially leading to image distortions, artifacts in electrical recordings, peripheral nerve stimulation, and heating of metallic implants • Fast gradient switching leads to loud acoustic noise, which impacts subject comfort and causes stress • Fast gradient switching leads to physical vibrations of the scanner, which can impact image quality, external recordings, and subject comfort • High gradient duty cycle can induce gradient heating	• Incremental gradient switching in zero-TE approaches leads to minimal eddy currents; minimal interference with electrical recordings was, for instance, demonstrated with MB-SWIFT^37^ • Incremental gradient switching in zero-TE approaches enables quiet or even nearly silent scanning,^33,35,37^ with great benefits for subject comfort and stress reduction • Incremental gradient switching in zero-TE approaches leads to minimal physical vibrations of the scanner • Incremental gradient switching holds for zero-TE approaches, not for UTE • Gradient duty cycle is generally high, and it can induce gradient heating when high BWs are used
Multimodal compatibility	• Limited compatibility with external probes and electrical recordings, primarily due to strong implant-induced susceptibility artifacts and strong interferences originating from eddy currents; safety of patient devices (e.g., implanted electrodes) can be a concern, especially because eddy currents may cause heating of the device	• Straightforward compatibility with external probes and electrical recordings,^28,38–40^ due to minimal implant-induced susceptibility artifacts and, for zero-TE methods, minimal eddy currents; safety of patient devices needs to be carefully evaluated, especially because high RF energy may cause heating of the device
**B. Specific characteristics relevant for fMRI implementations**		
Origin of functional contrast	• Sensitization to blood oxygenation (BOLD contrast)^1,2^ • Sensitization to CBF9 • Sensitization to CBV^12^ • Functional contrasts well known and characterized thanks to decades of research	• Inflow-mediated T_1_ sensitization to CBF/CBV using local RF transmit and/or slab selection^28,41^ • Likely minor T_1_ sensitization to molecular oxygen • Other cellular-specific T_1_ contributions possible but likely negligible • Origins of FID-based functional contrasts currently understood, but relative contributions are still being characterized in detail with focused efforts
Sensitivity and specificity to neuronal activity	• BOLD, CBF, and CBV functional contrasts are sensitive and specific proxies of neuronal activity, especially at ultrahigh magnetic fields (for reviews, see Refs. ^8,10,11,13^)	• FID-based functional contrast proven to be a reliable proxy of neuronal activity, at least for zero-TE fMRI^41^ • Sensitivity of FID-based fMRI increases with B_0_^42^ • Functional SNR, as determined by typical t-scores of activated areas, is comparable, if not superior, to that obtained with BOLD fMRI^27,28,35,42^
Hardware requirements for fMRI	• Require strong/fast gradients to achieve high spatial temporal resolution • Require dedicated B_0_ shimming, especially at high and ultrahigh fields • No need of local transmit RF coils for contrast sensitization • No need of ultrafast T/R switching • No need of special equipment materials	• No need of strong/fast gradients to achieve high spatial temporal resolution • No need of dedicated B_0_ shimming • May require local transmit RF coils for sensitizing the contrast to inflow • May require ultrafast T/R switching for minimizing acquisition delays and loss of initial FID points • May require special equipment materials (e.g., polytetrafluoroethylene) to prevent confounding signals originating from plastic components
Acquisition strategies for fMRI	• Acquisition strategies optimized thanks to decades of development • Many strategies available for acquisition acceleration, and continuously being developed (e.g., multiband, parallel imaging)	• Optimization of acquisition strategies (e.g., k-space trajectories, number of spokes, flip angle, bandwidth, oversampling) is an ongoing focus of research • Acceleration strategies, such as parallel imaging, possible but not used in fMRI yet
Image reconstruction and fMRI processing	• Typically Cartesian reconstructions, commonly available, and constantly evolving • Many tools available for fMRI processing of EPI time series	• Non-Cartesian reconstructions, commonly available; optimization for fMRI is an area of development • Available tools for fMRI processing may be suboptimal for zero/ultrashort TE images, which have a different contrast from the EPI images
Spatiotemporal resolution	• Spatial resolution up to mesoscale, also with full-brain coverage^25^ • Temporal resolution typically 0.5–2 s, and possibly down to 100 ms^23^ • BOLD-fMRI provides snapshots of both hemodynamic response and physiological status every TR • Sampling of physiological confounds (e.g., heart beats, vascular pulsations) may be challenging especially in rodents	• Spatial resolutions typical for fMRI straightforward; in FID-based approaches, meso-scale resolution is more difficult, as slab selection is not possible, and thus the whole FOV needs to be encoded • When number of spokes is reduced to shorten volume TR, the radial acquisition may lead to image blurring due to apodization performed during gridding of the under-sampled k-space periphery • fMRI volume collection typically uses 1000–2000 spokes, and volume TRs are typically 0.5–2.0 s; temporal resolutions up to 100 ms and less are possible with retrospective rebinning (EVER-SWIFT)^41,43^ • Volume TRs are similar to those of EPI-based fMRI, resulting in similar sampling rate of the hemodynamic response • Spoke TRs < 1 ms enable virtually continuous insights (i.e., with > 1 kHz sampling rate) of the physiological status during the volume TR, possibly allowing detection of heart beat and breathing-induced effects, and even imaging of vascular pulsations by using spoke reordering^44^
SAR ∝ n * FA^2^ * BW,^45^ where *n* = # of pulses during volume TR, FA = flip angle, BW = 1/T_p_ (with T_p_ = pulse duration)	• The several tens of 50°-70° excitation pulses typically used during a volume TR for full-brain coverage are generally not a challenge for SAR, given their typically low excitation BW (0.2–1 kHz, T_p_ = 1–5 ms) • SAR generally lower than comparable FID-based fMRI acquisitions	• The few thousand (typically 2000) 2°-3° excitation pulses typically used during a volume TR for full-brain fMRI coverage can be a challenge for SAR, given their very high excitation BW (100–200 kHz, T_p_ = 5–10 μs) • BW can still be lowered to find tradeoffs and handle SAR • SAR of UTE generally lower than zero-TE fMRI, due to lower BW (e.g., 50 kHz, T_p_ = 20 μs). UTE-fMRI already applied safely at 7 T^46,47^
**C. Progress of fMRI implementations**		
Initial fMRI demonstration in vivo	• 1992—BOLD contrast^3–5^ • 1994—CBF contrast^9^ • 2003—CBV contrast^12^	• 2012—SWIFT^27^ • 2017—MB-SWIFT^28^ • 2024—ZTE (with in-flow contrast)^48^ • 2024—UTE^47^ • 2025—SORDINO^35^
Mature fMRI applications	• Task-based and resting-state fMRI • Studies in humans, non-humate primates, and rodents • Multicenter studies	• Task-based and resting state fMRI in rodents • Awake studies in rodents
Emerging fMRI applications	• Big-data fMRI • Mesoscale fMRI • fMRI of cortical layers • fMRI of cortical columns	• fMRI beyond the brain: nose, spinal cord • Comprehensive CNS fMRI with dual-FOV fMRI • Social fMRI • Sleep fMRI • Awake behaving animals • Neurofluids beyond CBF • Sensory and cognitive studies without impact of loud EPI acoustic noise
fMRI use at ultrahigh magnetic field	• Common up to 7 T for humans and 9.4 T for animals but generally challenging due to sensitivity to B_0_ inhomogeneities • Artifacts originating from B_0_ inhomogeneities cannot always be recovered	• Straightforward at any field strength due to minimal sensitivity to B_0_ inhomogeneities • Image distortions originating from B_0_ inhomogeneities can be minimized especially with zero-TE approaches
Clinical translation	• Fully implemented on clinical scanners with vendor-released hardware and software • Many tools publicly available for acquisition and processing • Harmonized acquisition protocols and processing pipelines, developed over years of extensive collective efforts (i.e., HCP^6^)	• Zero-TE fMRI implemented so far only on research^27^ or clinical^49^ scanners with custom software and hardware • UTE-fMRI implemented so far only at 7 T,^46,47^ with custom software • Limited shared resources for both acquisition and processing; in fact, optimization of acquisition parameters is an active area of on-going research • Trade-offs among the different FID-based approaches need to consider the application, available hardware, the targeted spatiotemporal resolutions, and tolerance to artifacts; for instance, if ultrafast T/R switches are not available such as on clinical scanners, one can still use relatively lower excitation BWs, at the expense of reducing the resilience to B_0_ inhomogeneities • UTE is more flexible with vendor-provided head RF coils than zero-TE approaches, because it enables slab selection for inflow sensitization; however, UTE is acoustically noisy, and it typically leads to smaller excitation BWs than zero-TE sequences • Given the multiple frequency excitations during each readout gradient, MB-SWIFT is more flexible than ZTE for achieving efficient signal averaging and for power management to obtain high BWs; MB-SWIFT becomes ZTE by adjusting sequence parameters

Abbreviations: 2D, two-dimensional; 3D, three-dimensional; BOLD, blood oxygen level -dependent; BW, bandwidth; CBF, cerebral blood flow; CBV, cerebral blood volume; CNS, central nervous system; EPI, echo planar imaging; FA, flip angle; FID, free induction decay; fMRI, functional MRI; FOV, field of view; GRE, gradient recalled echo; MB-SWIFT, multiband sweep imaging with Fourier transformation; RF, radiofrequency; SAR, specific absorption rate; SE, spin-echo; SNR, signal-to-noise ratio; TR, repetition time; T/R, transmit/receive; UTE, ultrashort echo time; ZTE, zero echo time.

## Data Availability

Data sharing not applicable to this article as no datasets were generated or analysed during the current study.
